# Supplementary material for: Contributions of Microelectrochemical Scanning Techniques for the Efficient Detection of Localized Corrosion Processes at the Cut Edges of Polymer-Coated Galvanized Steel
Source: Molecules. 2022 Mar 27;27(7):2167. doi: 10.3390/molecules27072167 (PMC9000633; doi:10.3390/molecules27072167)
Supplement: Supplementary file 1 [file molecules-27-02167-s001.zip › molecules-1649435-supplementary.pdf]

molecules

MDPI

Article

Contributions of Microelectrochemical Scanning Techniques for the Efficient Detection of Localized Corrosion Processes at the Cut Edges of Polymer-Coated Galvanized Steel

Dániel Filotás [1,2,*], Javier Izquierdo [3,4], Bibiana M. Fernández-Pérez [3], Lívia Nagy [1,2], Géza Nagy [1,2] and Ricardo M. Souto [3,4,*]

1 Department of General and Physical Chemistry, Faculty of Sciences, University of Pécs, Ifjúság útja 6, 7624 Pécs, Hungary; lnagy@ttk.pte.hu (L.N.); g-nagy@gamma.ttk.pte.hu (G.N.)
2 János Szentágothai Research Center, University of Pécs, Ifjúság u. 20, 7624 Pécs, Hungary
3 Department of Chemistry, Universidad de La Laguna, P.O. Box 456, 38200 La Laguna, Tenerife, Spain; jizquier@ull.edu.es (J.I.); bfernand@ull.edu.es (B.M.F.-P.)
4 Institute of Material Science and Nanotechnology, Universidad de La Laguna, 38200 La Laguna, Tenerife, Spain
* Correspondence: filotasdaniel@gmail.com (D.F.); rsouto@ull.es (R.M.S.)

Figure S1. Sequence of SVET maps recorded on the cut edge of galvanized steel without organic coating layers during immersion in 1 mM NaCl solution

The X-Y dimensions of the maps are 1580 × 6000 μm^2, while the coloring bars give the current density values expressed in μA cm^{-2}.

15 min

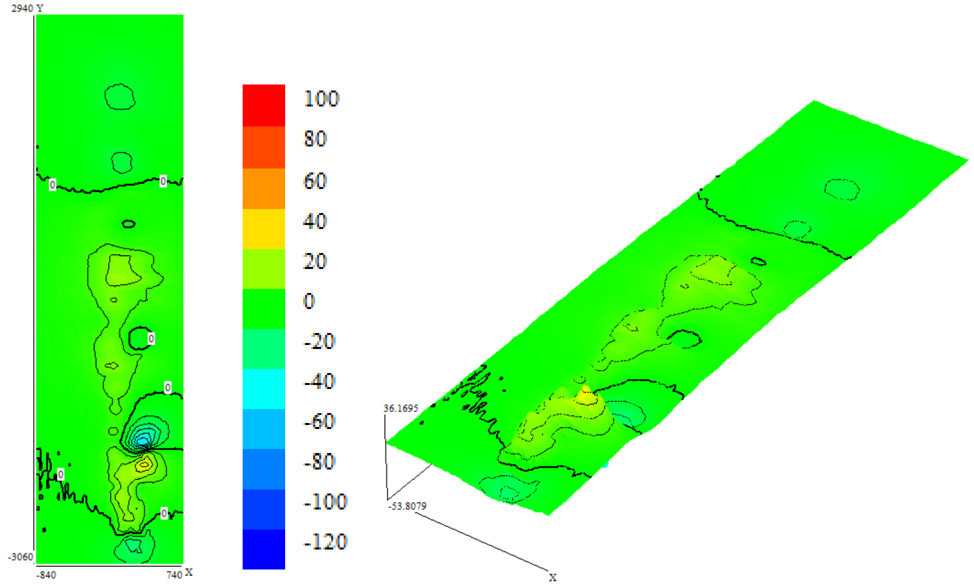

30 min

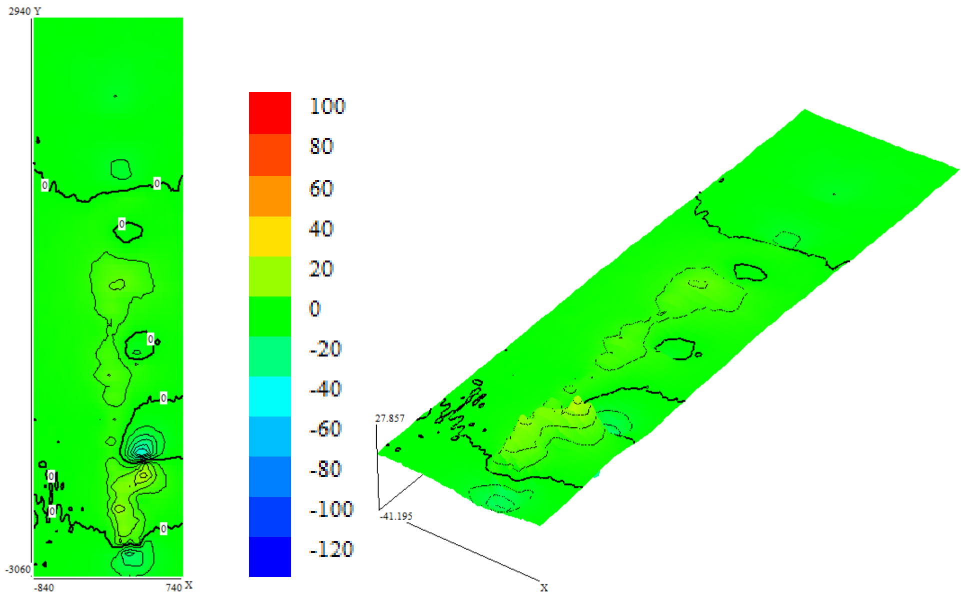

45 min

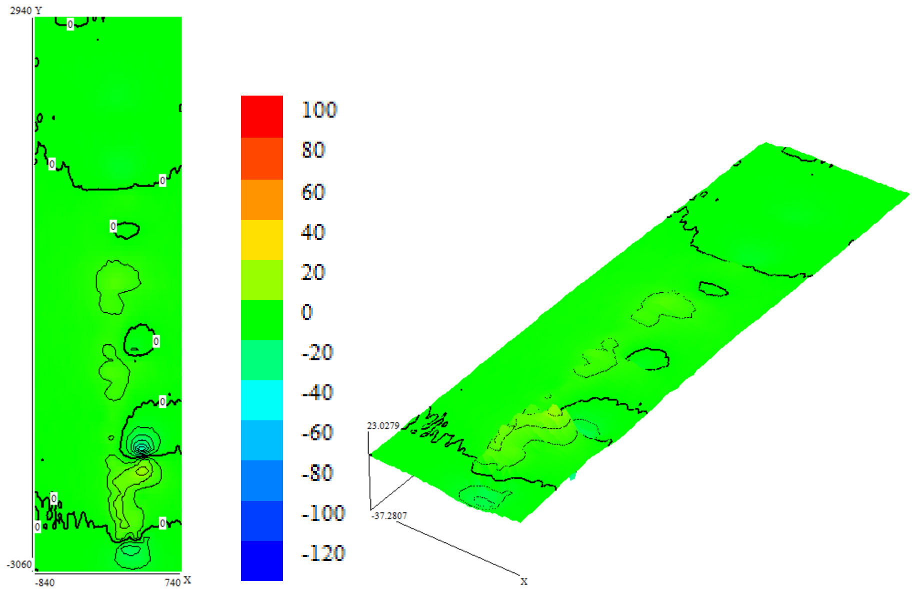

60 min

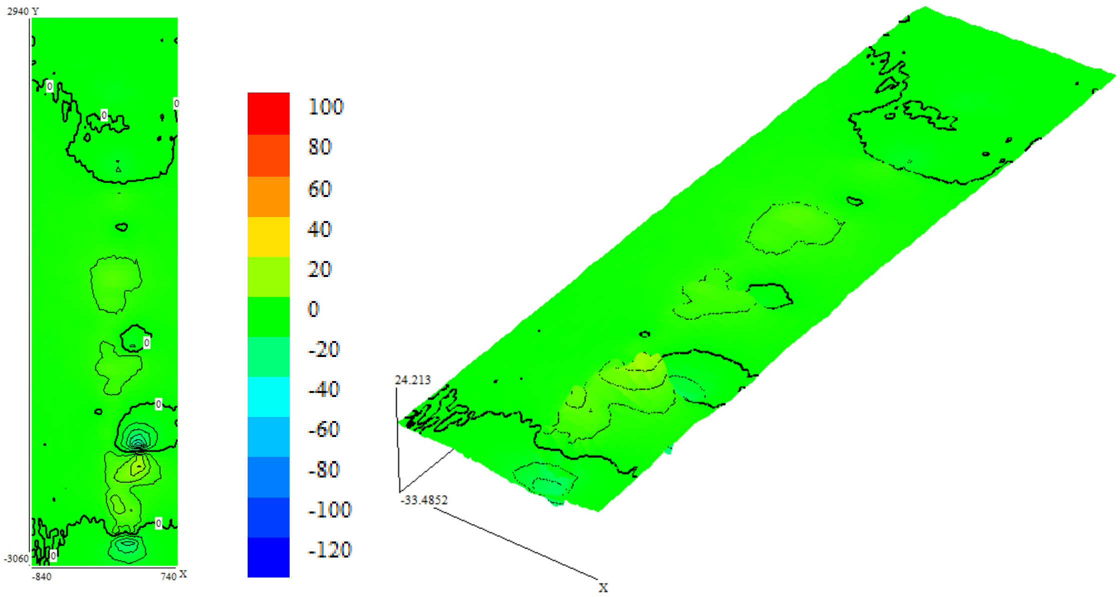
2940 Y
-3060
-840
740 X
100
80
60
40
20
0
-20
-40
-60
-80
-100
-120
24.213
-33.4852
x

75 min

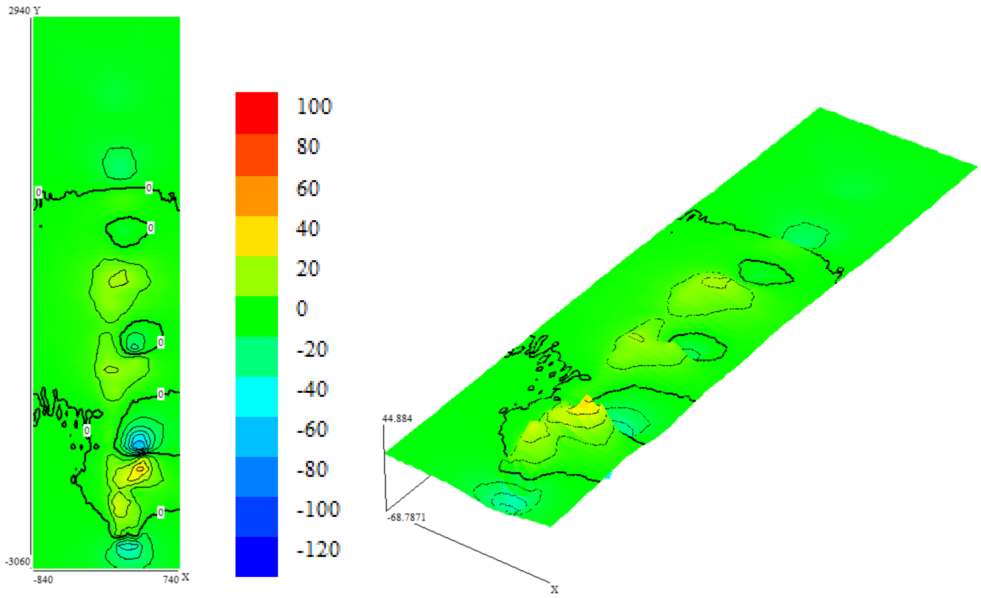
2940 Y
-3060
-840
740 X
100
80
60
40
20
0
-20
-40
-60
-80
-100
-120
44.884
-68.7871
x

90 min

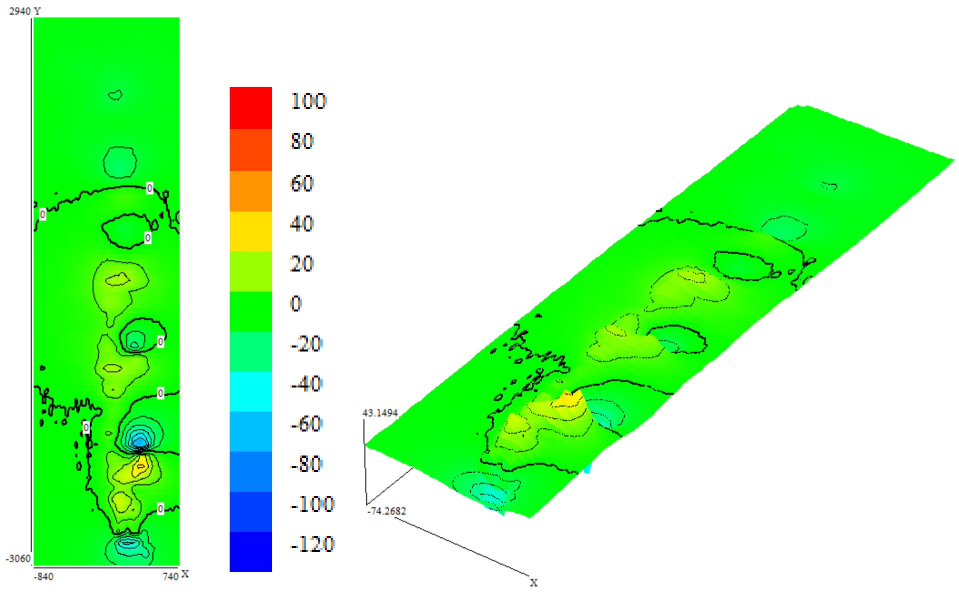

105 min

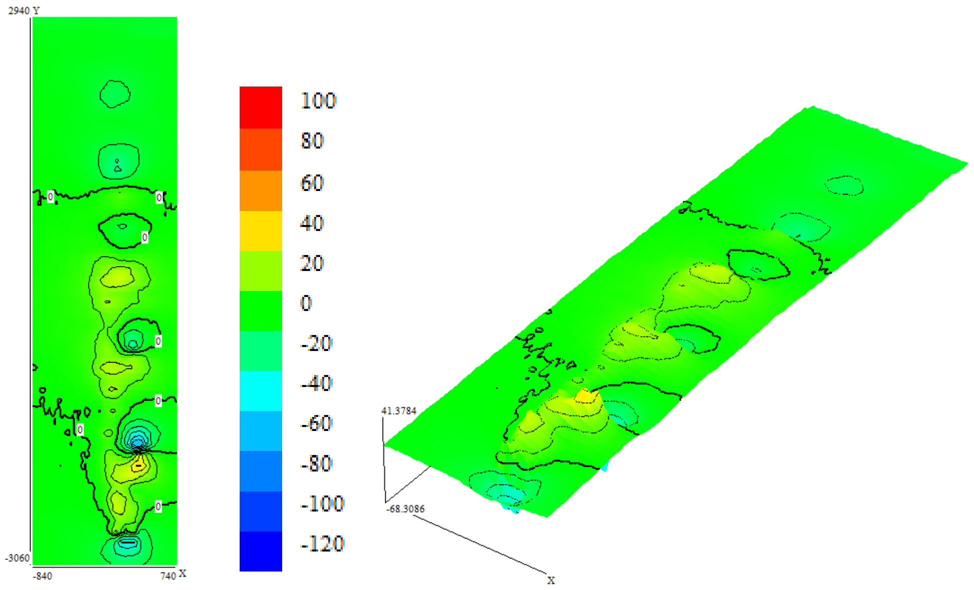

120 min

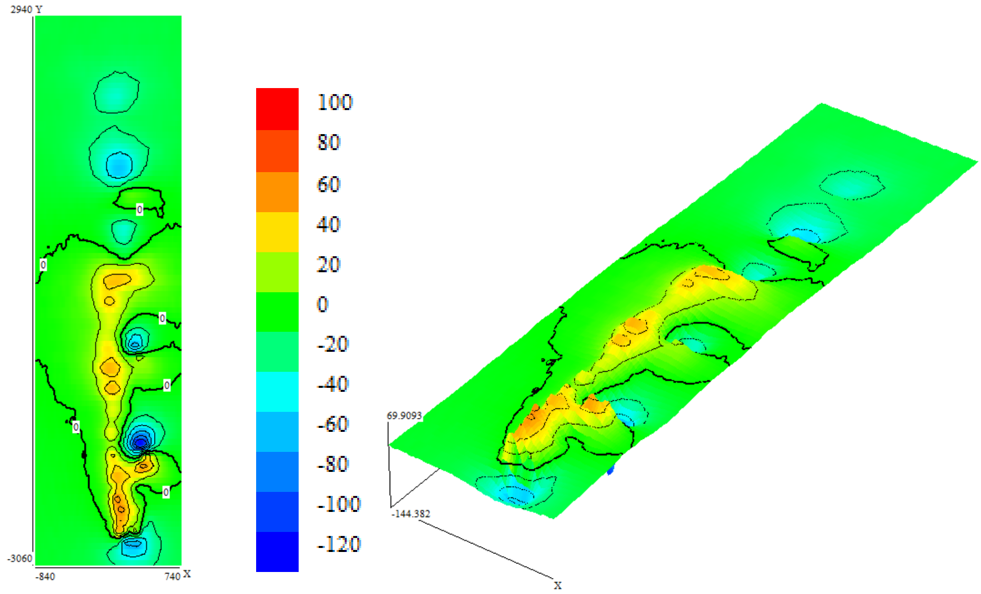

135 min

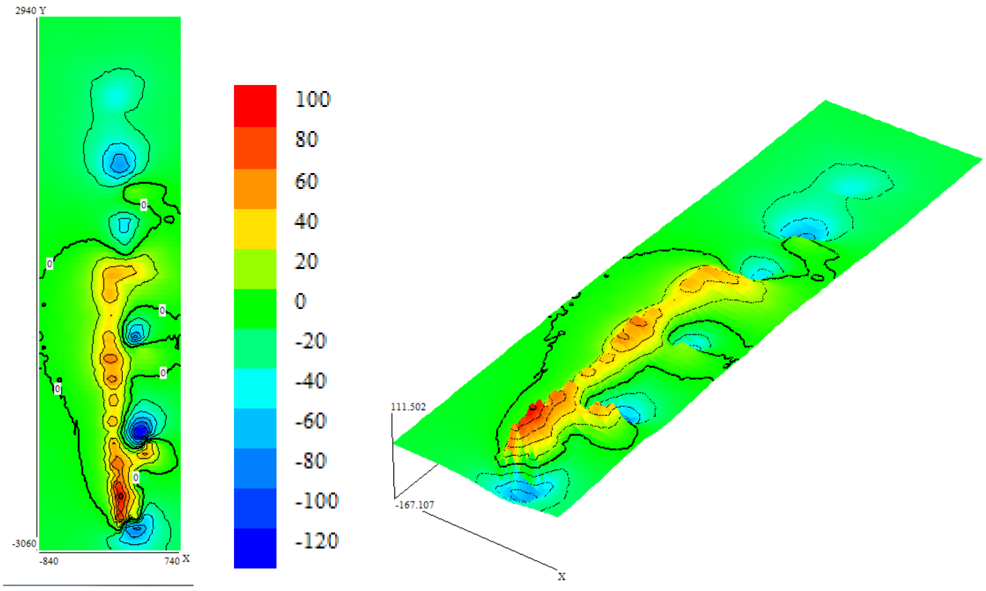

150 min

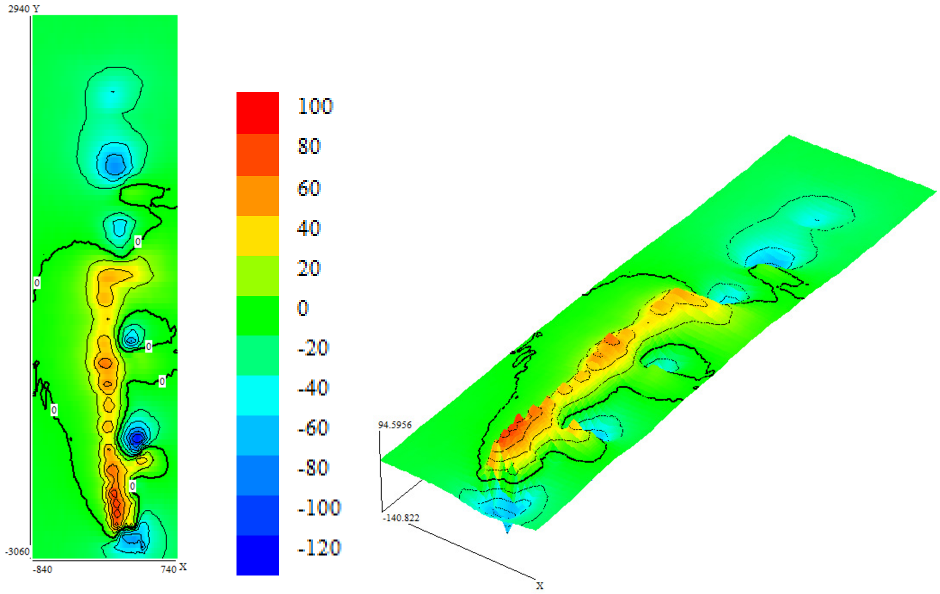
2940 Y
-3060
-840
740 X
100
80
60
40
20
0
-20
-40
-60
-80
-100
-120
94.5956
-140.822
X

165 min

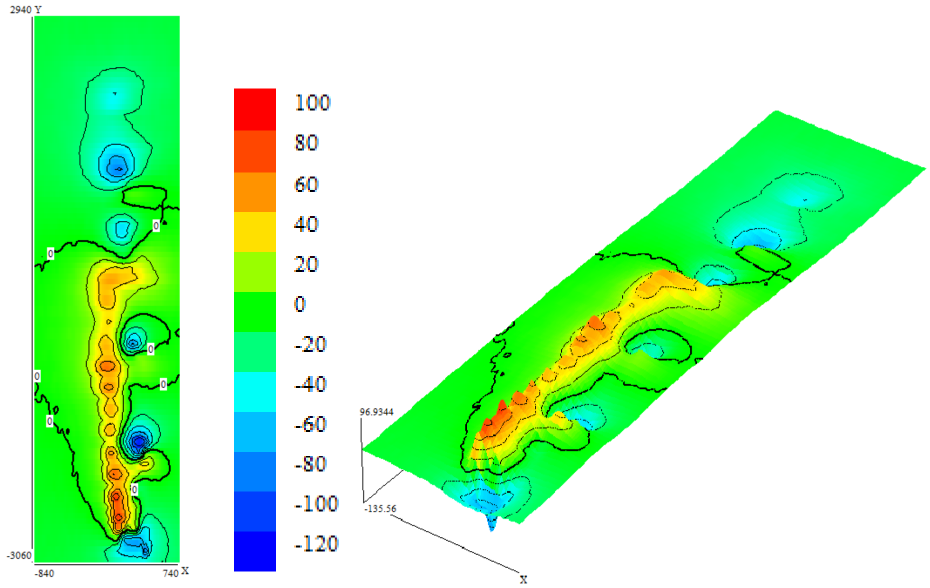
2940 Y
-3060
-840
740 X
100
80
60
40
20
0
-20
-40
-60
-80
-100
-120
96.9344
-135.56
X

Figure S2. Sequence of SVET maps recorded on the cut edge of the complete coil coating system during immersion in 1 mM NaCl solution

The X-Y dimensions of the maps are 1010 × 2790 μm², while the coloring bars give the current density values expressed in μA cm⁻².

15 min

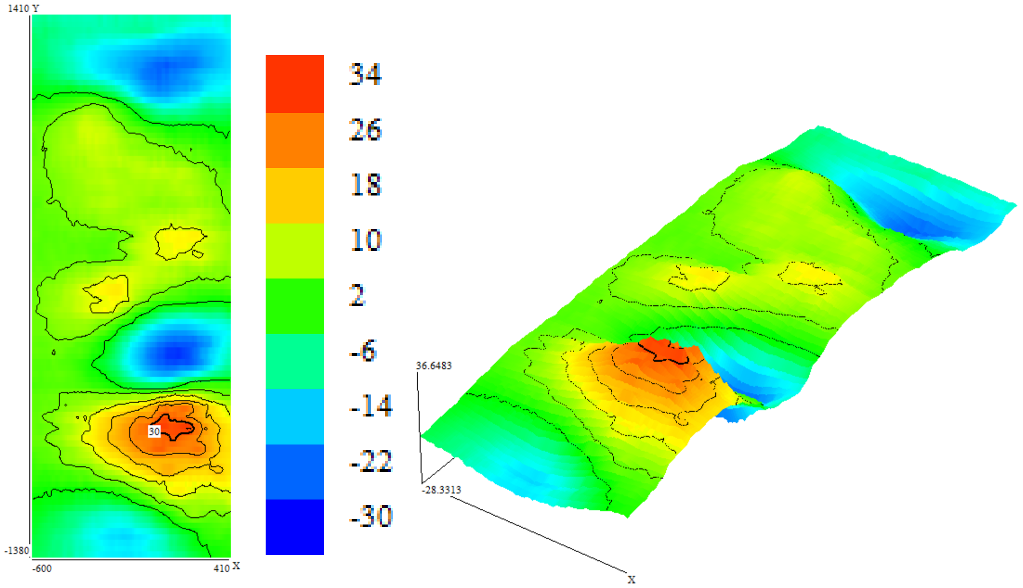

30min

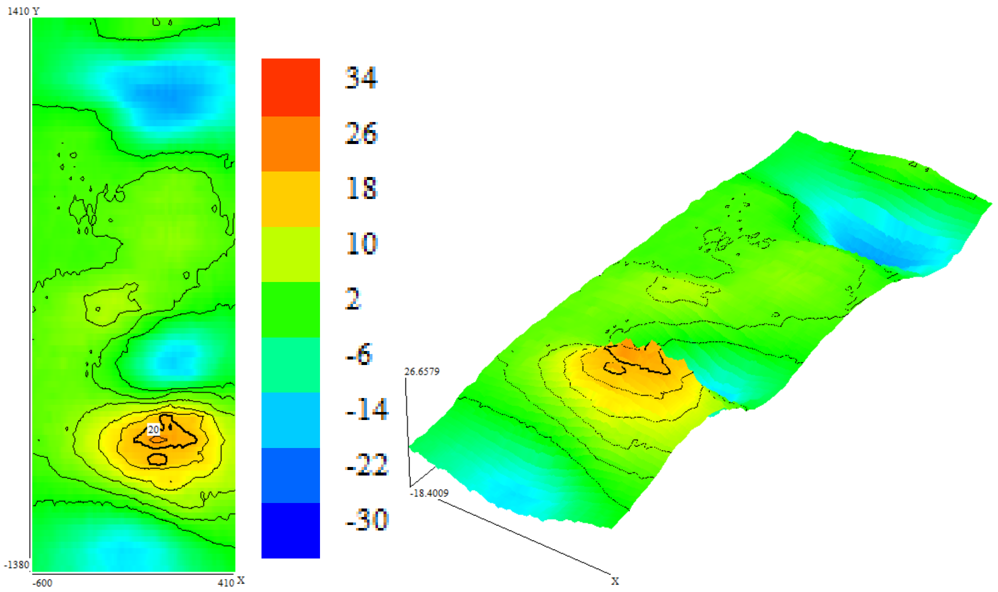

45 min

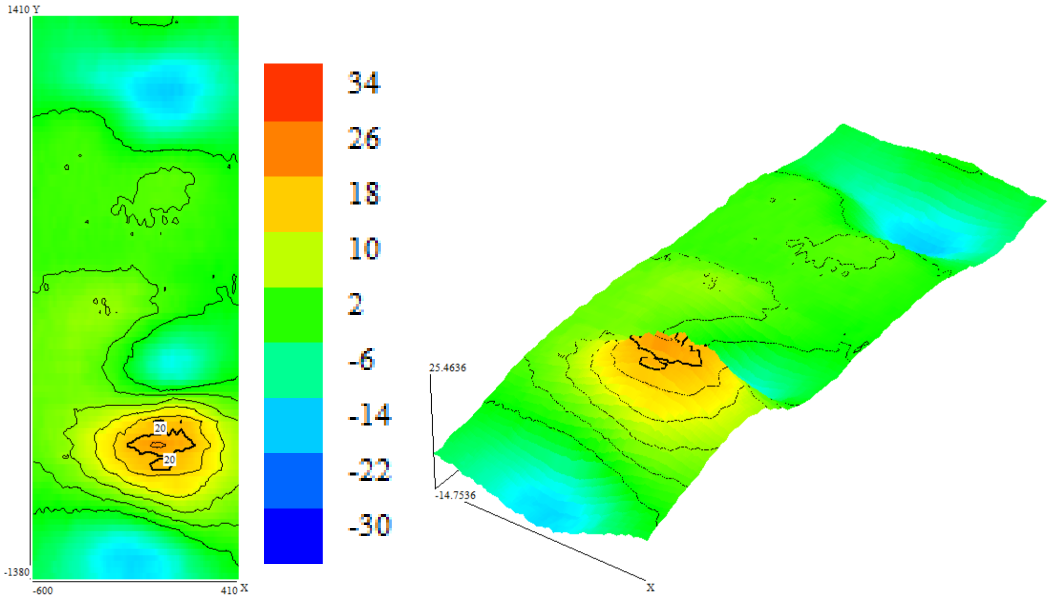
1410 Y
20
20
-1380
-600
410 X
34
26
18
10
2
-6
-14
-22
-30
25.4636
-14.7536
X

60 min

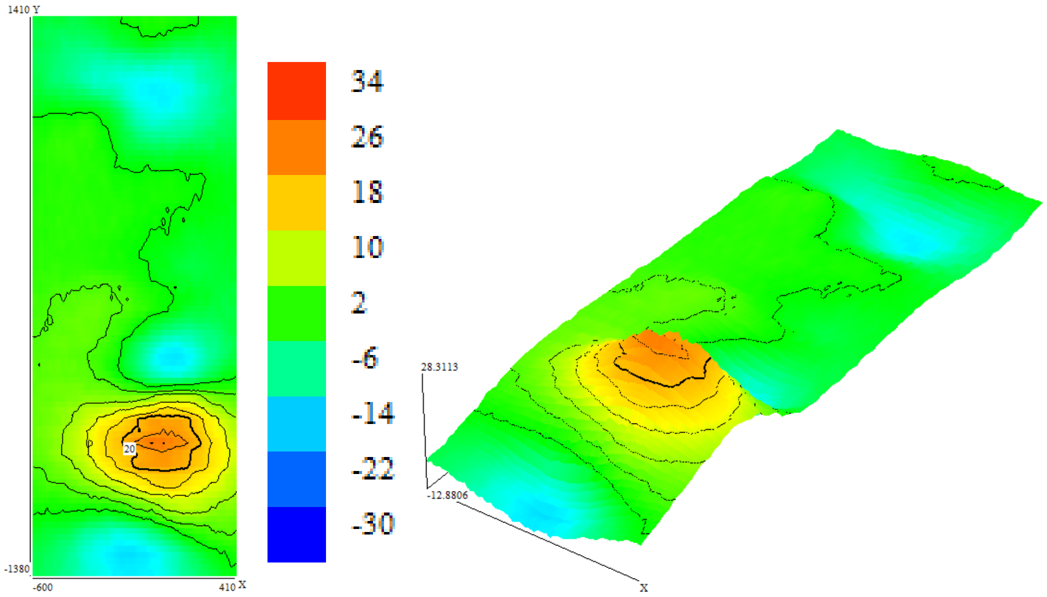
1410 Y
20
-1380
-600
410 X
34
26
18
10
2
-6
-14
-22
-30
28.3113
-12.8806
X

75 min

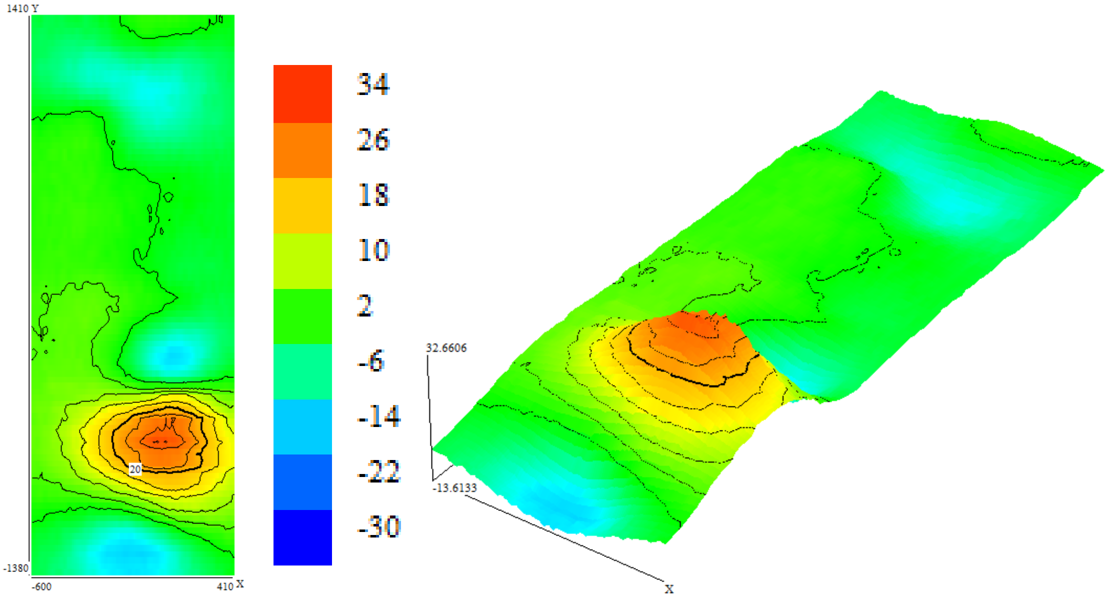

90 min

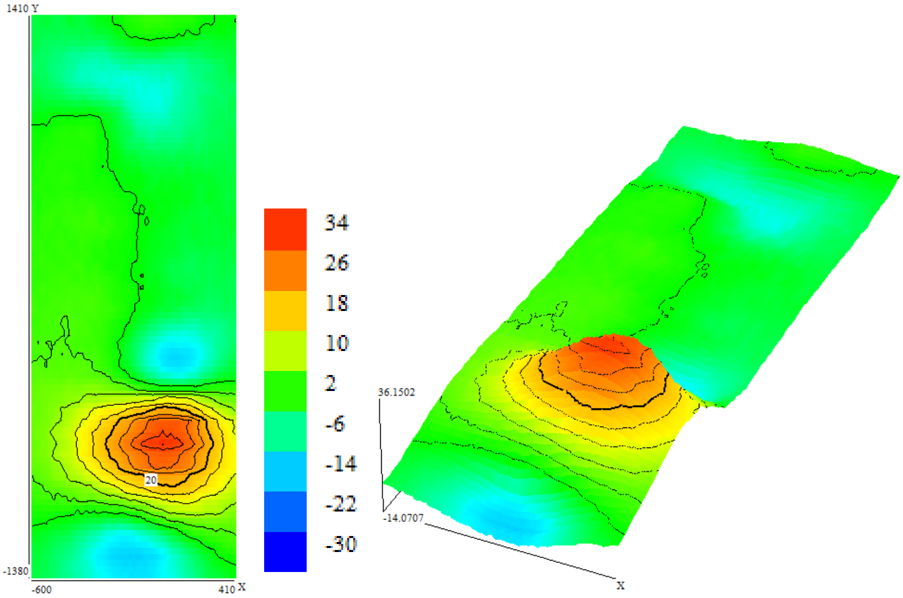

105 min

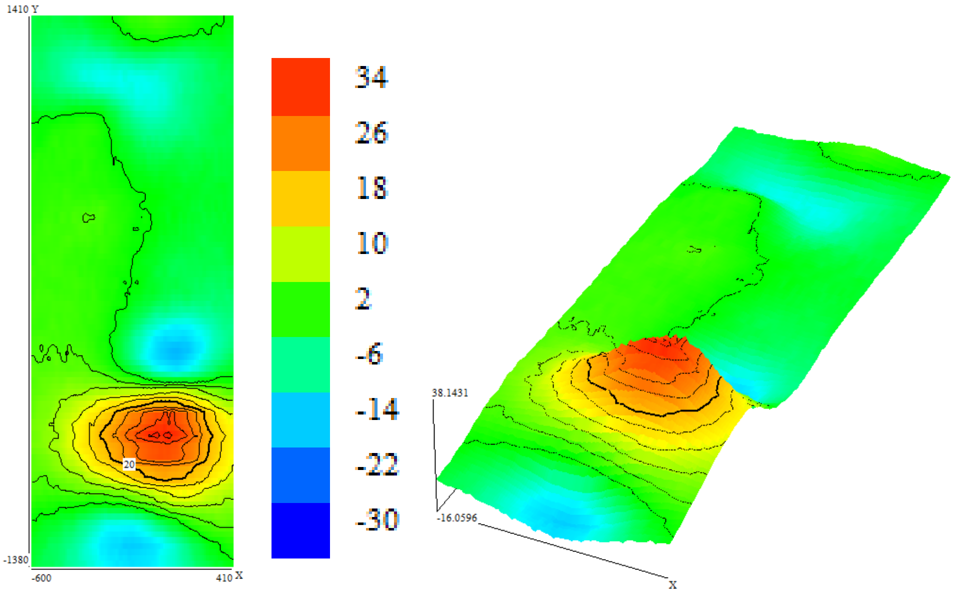

120 min

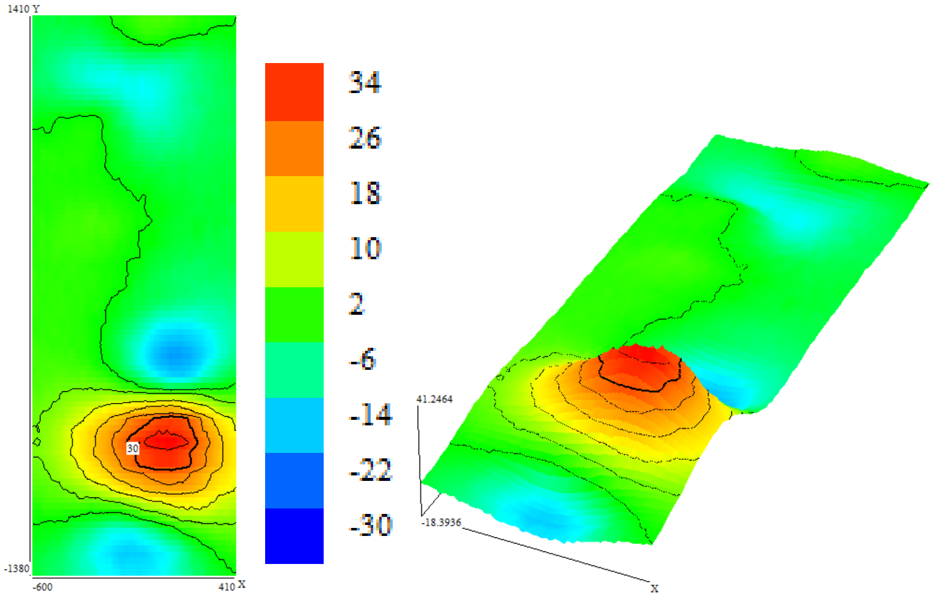

135 min

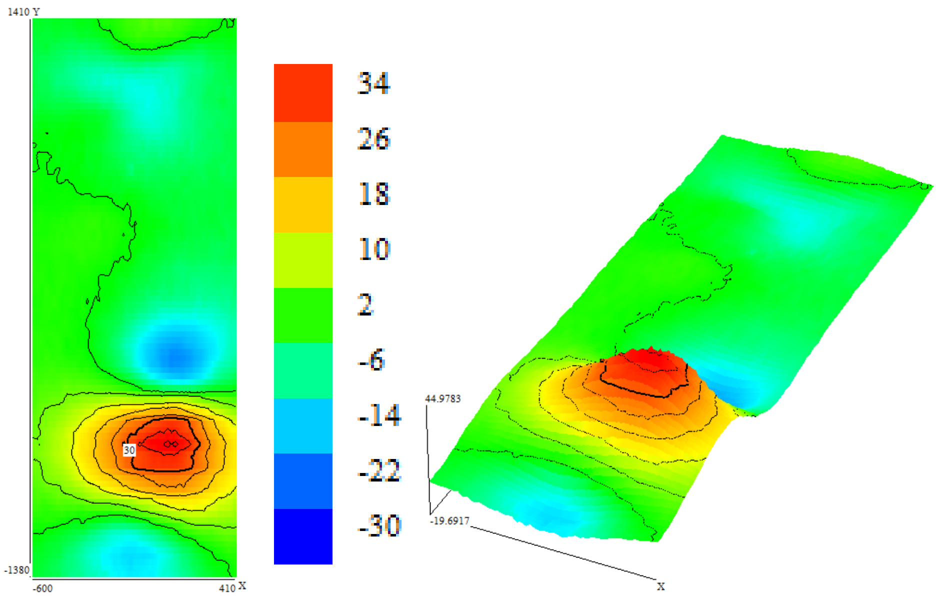

150 min

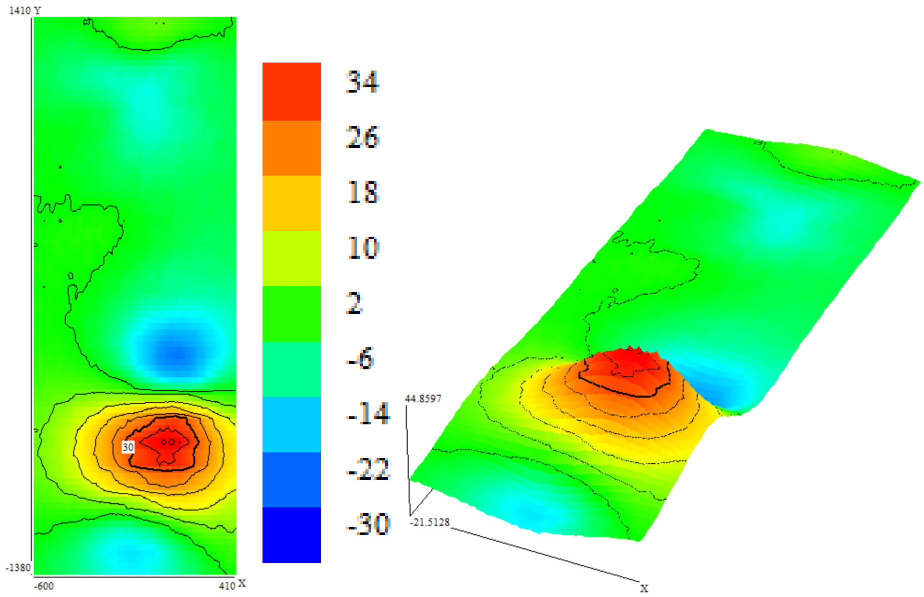

165 min

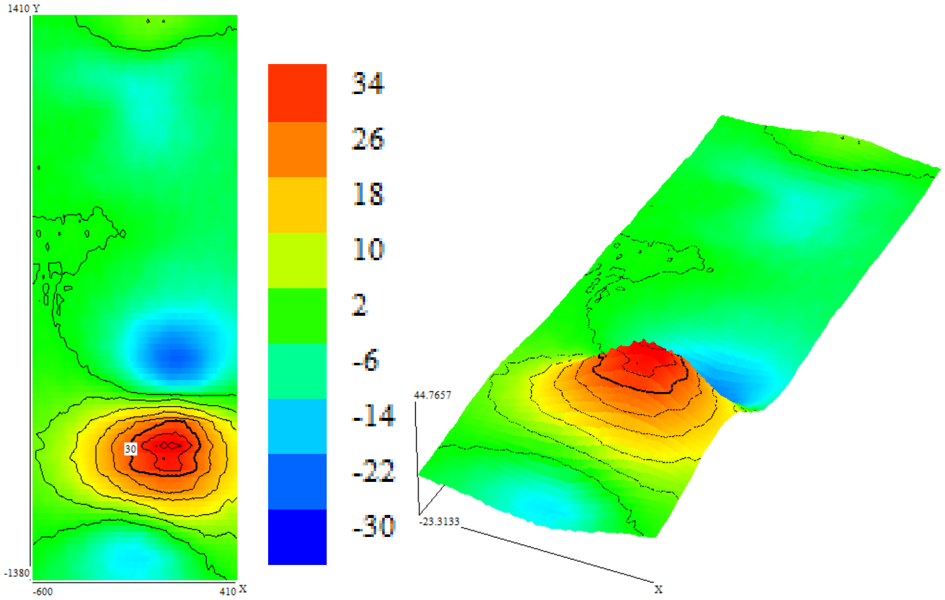
1410 Y
34
26
18
10
2
-6
-14
-22
-30
30
-1380
-600
410 X
44.7657
-23.3133
X

180 min

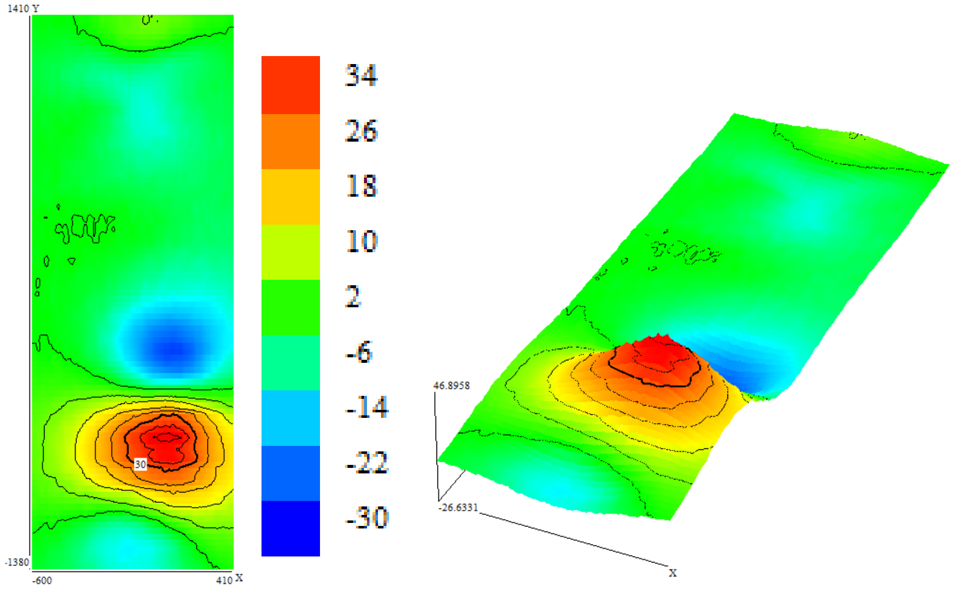
1410 Y
34
26
18
10
2
-6
-14
-22
-30
30
-1380
-600
410 X
46.8958
-26.6331
X
